# Protective effect of prebiotic and exercise intervention on knee health in a rat model of diet-induced obesity

**DOI:** 10.1038/s41598-019-40601-x

**Published:** 2019-03-07

**Authors:** Jaqueline Lourdes Rios, Marc R. Bomhof, Raylene A. Reimer, David A. Hart, Kelsey H. Collins, Walter Herzog

**Affiliations:** 1 Human Performance Laboratory, Faculty of Kinesiology, University of Calgary, Calgary, AB Canada; 2 McCaig Institute for Bone and Joint Health, University of Calgary, Calgary, AB Canada; 3 Department of Kinesiology & Physical Education, University of Lethbridge, Lethbridge, AB Canada; 4 Department of Biochemistry and Molecular Biology, University of Calgary, Calgary, AB Canada

## Abstract

Obesity, and associated metabolic syndrome, have been identified as primary risk factors for the development of knee osteoarthritis (OA), representing nearly 60% of the OA patient population. In this study, we sought to determine the effects of prebiotic fibre supplementation, aerobic exercise, and the combination of the two interventions, on the development of metabolic knee osteoarthritis in a high-fat/high-sucrose (HFS) diet-induced rat model of obesity. Twelve-week-old male Sprague-Dawley rats were randomized into five groups: a non-exercising control group fed a standard chow diet, a non-exercising group fed a HFS diet, a non-exercising group fed a HFS diet combined with prebiotic fibre supplement, an exercise group fed a HFS diet, and an exercise group fed a HFS diet combined with prebiotic fibre supplement. Outcome measures included knee joint damage, percent body fat, insulin sensitivity, serum lipid profile, serum endotoxin, serum and synovial fluid cytokines and adipokines, and cecal microbiota. Prebiotic fibre supplementation, aerobic exercise, and the combination of the two interventions completely prevented knee joint damage that is otherwise observed in this rat model of obesity. Prevention of knee damage was associated with a normalization of insulin resistance, leptin levels, dyslipidemia, gut microbiota, and endotoxemia in the HFS-fed rats.

## Introduction

Obesity, and associated metabolic syndrome, have been identified as primary risk factors for the development of knee osteoarthritis (OA)^1,2^. OA linked to obesity has been defined as a separate “metabolic OA” phenotype^3^, and this phenotype represents nearly 60% of the OA patient population^4^. Metabolic OA is associated with large visceral fat depots deposits^5^ that release inflammatory cytokines/adipokines resulting in low-level systemic inflammation^6,7^. Exposing rats to a high-fat/high-sucrose (HFS) diet leads to dysbiosis (impaired gut microbiota)^8^, increased body fat, systemic inflammation, knee joint inflammation^9^, intramuscular fat deposition^8,10^, and knee damage in rats within 12-weeks^9^ (for a detailed review see Collins *et al*.^11^, and Courties *et al*.^2^).

In many cases, obesity is considered a modifiable risk factor^12^. Exercise is known as an effective modulator of body fat, systemic inflammation, and blood lipid levels^13–16^. Similarly, dietary prebiotic fibre supplementation has been shown to decrease body fat and increase metabolic health in rodents^17,18^ and humans^19^. For example, exercise has been suggested to enhance the ability of skeletal muscles to use lipids, thus allowing for better control of plasma lipid levels^13^; while the fermentation of oligofructose (OFS) prebiotic fibre, a non-digestible plant-derived carbohydrate^20^, by the gut microbiota leads to the production of short-chain fatty acids (SCFA) in the colon, which bind to fatty acid receptors and stimulate hormones associated with a reduction in appetite, delay of gastric emptying, and improvement of insulin sensitivity^21,22^. However, there are no studies to date that have examined the effects of exercise and dietary intervention in the knee joint during obesity development (spontaneous knee OA in the metabolic OA phenotype). We hypothesize that prebiotic fibre supplementation, an aerobic exercise regime, and the combination of prebiotic fibre and aerobic exercise will prevent OA-like damage in the knee of rats fed a HFS diet by controlling inflammation, dysbiosis, and metabolic syndrome.

In this study, we determined the effects of prebiotic fibre supplementation, aerobic exercise, and the combination of prebiotic fibre and aerobic exercise on the development of metabolic knee OA in a HFS diet-induced rat model of obesity. Our findings, confirmed our hypotheses, indicating that prebiotic fibre, aerobic exercise, and the combination of prebiotic fibre and aerobic exercise prevented the development of OA-like damage that is observed in rats on a HFS diet that are not receiving these interventions. We defined OA-like damage as a disorder that results in structural changes to joint integrity involving cartilage, subchondral bone, menisci, and/or synovium.

## Results

### Prebiotic fibre and aerobic exercise prevent OA-like damage to the knee of rats exposed to a HFS diet

**Figure 1:**
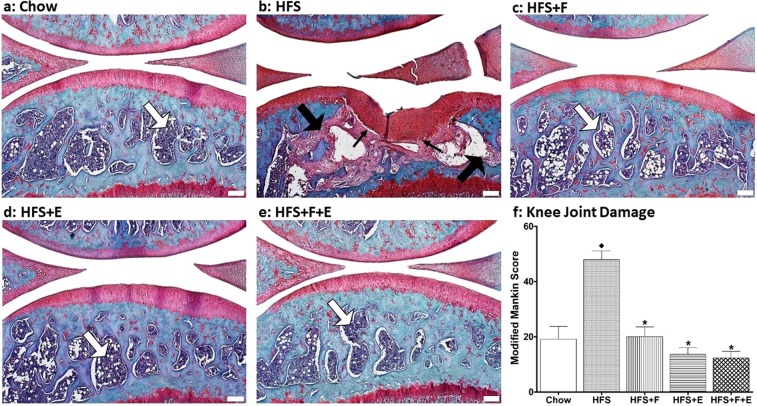
Knee joint integrity. White thick arrows indicate healthy bone marrow. Black thick arrows indicate that the normal marrow has been replaced with a fibrotic marrow. Black thin arrows indicate that the fibrous and granulation tissue extended to the subchondral bone and to the tidemark. ^♦^Indicates difference from chow group, while *Indicates difference from HFS group. Chow: chow control diet; HFS: high fat/high sucrose diet; F: prebiotic fibre; E: aerobic exercise. Values are means ± 1 SEM (white line, bar = 200 µm).

The HFS obesity-inducing diet disrupts knee joint homeostasis of male Sprague Dawley rats resulting in structural damage to the joint (F_(4,51)_ = 22.387, p < 0.001). Our most prominent finding is that the subchondral bone in rats fed a HFS diet was replaced by a fibrotic tissue with consequent collapse of the adjacent cartilage, which is a novel contribution to the literature in this area (Fig. 1). The thick arrow on Fig. 1b indicates that normal bone has been replaced by fibrotic tissue in rats fed a HFS diet. The fibrous tissue extends to the tidemark in some places (Fig. 1b, thin arrow), and the loss of subchondral bone likely leads to the collapse of the bone and the adjacent cartilage. These changes were observed in the medial tibial plate, and medial/posterior femoral condyle of 75% (9 out 12) of rats fed a HFS diet. The other 3 rats presented changes in the subchondral bone, with thickening of the cartilage, but without the collapse (Supplementary Material Fig. 1). We have also observed thickening of the synovium in HFS fed rats when compare to chow rats (Supplementary Material Fig. 2).

**Figure 2:**
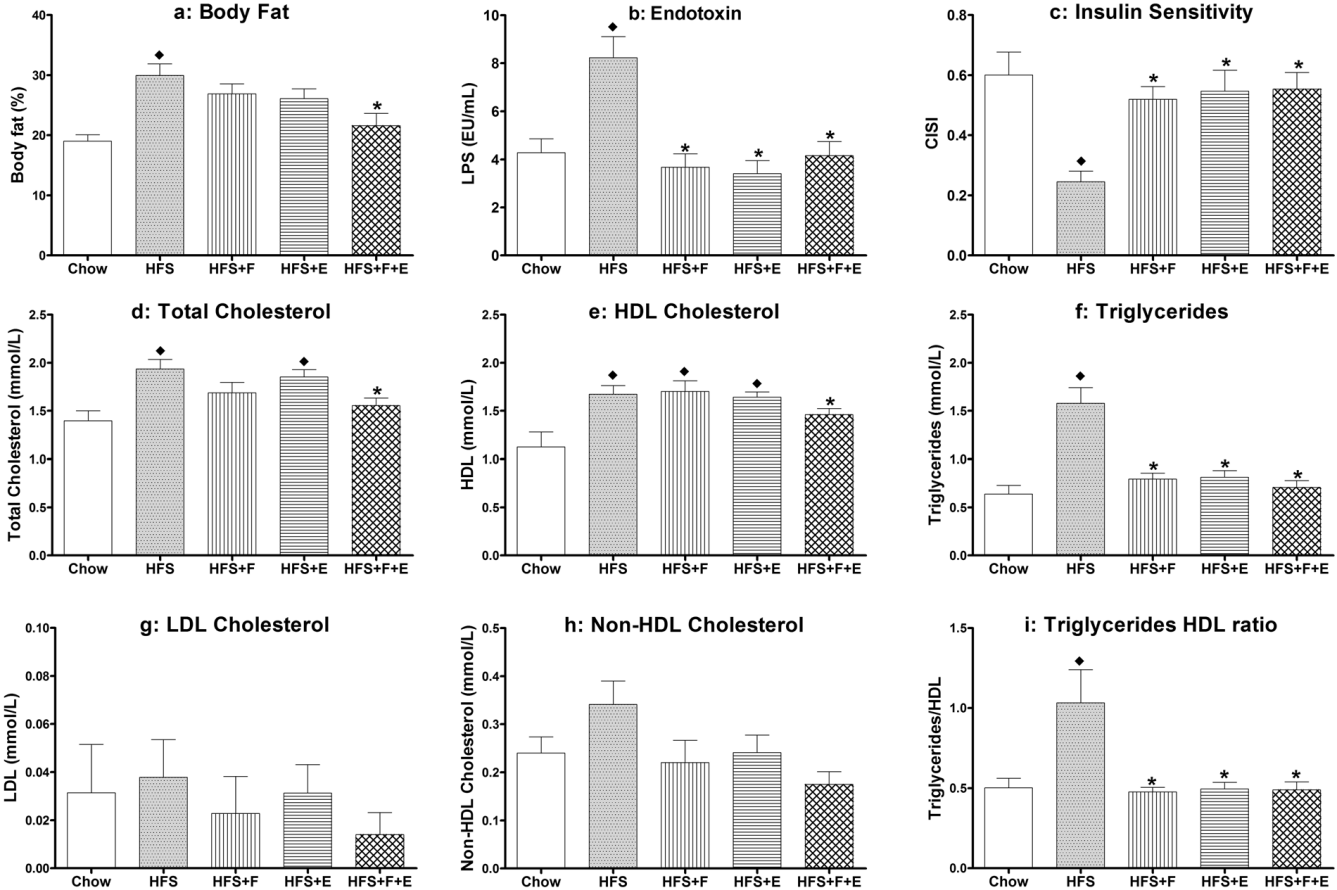
Metabolic profile. Body fat, endotoxin, composite insulin sensitivity index, serum lipid profile for rats in the chow, HFS, HFS + F, HFS + E, HFS + F + E groups at the end of the experimental protocol. ^♦^Indicates difference from chow group, while *indicates difference from HFS group. Chow: chow control diet; HFS: high fat/high sucrose diet; F: prebiotic fibre; E: aerobic exercise. Values are means ± 1 SEM.

We found that the prebiotic and exercise interventions, and the combination of the two, fully protected the knee from joint damage (all p < 0.001), despite the intake of a HFS diet (Fig. 1, Table 1).

**Table 1:** Knee joint damage, percent body fat, synovial fluid leptin, endotoxin, composite insulin sensitivity index, serum lipid profiles, and cecal microbiota for rats treated in the chow, HFS, HFS + F, HFS + E, HFS + F + E groups at the end of the experimental protocol.

	Chow	HFS	HFS + F	HFS + E	HFS + F + E	Two way Anova							One way Anova		
						F		E		F * E		Adjusted R^2^	p-value	partial η^2^	Observed Power
						p-value	partial η^2^	p-value	partial η^2^	p-value	partial η^2^				
Knee joint integrity	19.1 ± 4.7	47.9 ± 3.2	20.1 ± 3.5	13.6 ± 2.4	12.3 ± 2.4	**<0.001**	0.361	**<0.001**	0.539	**<0.001**	0.318	0.666	**<0.001**	0.637	1.000
Percent body fat, %	19.0 ± 1.1	29.9 ± 1.9	26.9 ± 1.6	26.1 ± 1.6	21.6 ± 2.0	**0.045**	0.088	**0.016**	0.125	0.690	0.004	0.141	**0.001**	0.299	0.962
SF: leptin, pg/mL	63.8 ± 8.5	251.8 ± 48.7	130.5 ± 20.7	125.4 ± 24.1	113.1 ± 18.3	**0.045**	0.092	**0.019**	0.125	0.098	0.064	0.188	**0.001**	0.304	0.959
Endotoxin, EU/ML	4.3 ± 0.6	8.2 ± 0.9	3.7 ± 0.6	3.4 ± 0.5	4.2 ± 0.6	**0.006**	0.161	**0.002**	0.200	**<0.001**	0.272	0.441	**<0.001**	0.432	0.999
CISI	0.60 ± 0.08	0.25 ± 0.04	0.52 ± 0.04	0.54 ± 0.07	0.56 ± 0.06	**0.011**	0.153	**0.003**	0.203	**0.016**	0.14	0.315	**0.001**	0.343	0.976
Total Cholesterol (mmol/L)	1.40 ± 0.10	1.94 ± 0.10	1.69 ± 0.11	1.85 ± 0.08	1.56 ± 0.08	**0.005**	0.179	0.244	0.033	0.792	0.002	0.156	**0.001**	0.36	0.956
HDL Cholesterol (mmol/L)	1.12 ± 0.16	1.67 ± 0.09	1.70 ± 0.11	1.64 ± 0.05	1.46 ± 0.06	0.359	0.019	0.108	0.058	0.208	0.036	0.044	**0.001**	0.297	0.96
LDL Cholesterol (mmol/L)	0.03 ± 0.02	0.04 ± 0.02	0.02 ± 0.02	0.03 ± 0.01	0.01 ± 0.01	0.230	0.033	0.567	0.008	0.930	**<**0.001	0.026	0.768	0.035	0.148
Non-HDL Cholesterol (mmol/L)	0.24 ± 0.03	0.34 ± 0.05	0.22 ± 0.05	0.24 ± 0.04	0.18 ± 0.03	**0.031**	0.160	0.091	0.102	0.512	0.016	0.183	0.052	0.248	0.67
Triglycerides (mmol/L)	0.64 ± 0.09	1.58 ± 0.16	0.79 ± 0.11	0.81 ± 0.07	0.71 ± 0.07	**<0.001**	0.310	**<0.001**	0.291	**0.002**	0.207	0.501	**<0.001**	0.543	1.000
Ratio: Total Chol/HDL	1.10 ± 0.03	1.17 ± 0.04	1.05 ± 0.01	1.13 ± 0.02	1.06 ± 0.02	**0.003**	0.195	0.545	0.009	0.345	0.022	0.158	**0.031**	0.198	0.741
Ratio: Tryg/HDL	0.50 ± 0.06	1.03 ± 0.21	0.48 ± 0.03	0.50 ± 0.04	0.49 ± 0.05	**0.022**	0.122	**0.032**	0.107	**0.025**	0.117	0.240	**0.002**	0.296	0.941
**Cecal Microbiota**															
*Bacteroides/Prevotella*	11.91 ± 1.48	4.26 ± 0.39	13.75 ± 2.46	5.95 ± 0.49	12.78 ± 2.26	**<0.001**	0.344	0.835	0.001	0.437	0.014	0.306	**<0.001**	0.346	0.988
*Bifidobacterium*	0.08 ± 0.02	0.26 ± 0.05	11.01 ± 1.91	0.21 ± 0.05	10.87 ± 1.40	**<0.001**	0.649	0.934	**<**0.001	0.973	**<**0.001	0.625	**<0.001**	0.680	1.000
*Enterobacteriaceae*	0.16 ± 0.03	0.85 ± 0.19	0.82 ± 0.13	0.52 ± 0.10	0.34 ± 0.08	0.412	0.015	**0.003**	0.178	0.551	0.008	0.139	**0.001**	0.292	0.956
*Lactobacillus*	3.65 ± 0.63	0.36 ± 0.11	3.07 ± 0.56	0.26 ± 0.07	2.37 ± 0.32	**<0.001**	0.552	0.224	0.033	0.368	0.018	0.532	**<0.001**	0.569	1.000
*C*. *coccoides* (cluster XIV)	7.20 ± 0.43	7.99 ± 0.63	8.07 ± 1.12	9.97 ± 0.67	8.03 ± 1.02	0.301	0.024	0.279	0.027	0.260	0.029	0.013	0.256	0.097	0.397
*C*. *leptum* (cluster IV)	14.08 ± 0.97	15.36 ± 1.70	3.49 ± 1.04	12.58 ± 1.14	4.27 ± 0.72	**<0.001**	0.615	0.410	0.015	0.147	0.047	0.598	**<0.001**	0.640	1.000
C. cluster I	1.23 ± 0.18	1.19 ± 0.24	0.50 ± 0.04	0.86 ± 0.07	0.44 ± 0.04	**<0.001**	0.296	0.141	0.049	0.309	0.024	0.285	**<0.001**	0.360	0.992
C. cluster XI	0.11 ± 0.01	0.30 ± 0.07	0.18 ± 0.05	0.28 ± 0.07	0.08 ± 0.02	**0.006**	0.157	0.264	0.028	0.499	0.010	0.128	**0.017**	0.208	0.810
*Roseburia*	0.66 ± 0.32	0.24 ± 0.08	1.98 ± 0.24	0.14 ± 0.05	1.91 ± 0.32	**<0.001**	0.624	0.679	0.004	0.936	**<**0.001	0.599	**<0.001**	0.577	1.000
*Methanobrevibacter*	0.01 ± 0.00	0.01 ± 0.00	**<**0.001	0.01 ± 0.00	0.00 ± 0.00	**<0.001**	0.592	0.980	**<**0.001	0.111	0.057	0.575	**<0.001**	0.594	1.000
*Akkemansia muciniphila*	1.08 ± 0.50	1.25 ± 0.26	0.21 ± 0.11	1.21 ± 0.25	0.14 ± 0.06	**<0.001**	0.407	0.786	0.002	0.934	**<**0.001	0.367	**0.001**	0.300	0.963
*Faecalibacterium prausnitzii*	0.31 ± 0.05	0.95 ± 0.23	0.21 ± 0.08	0.52 ± 0.11	0.23 ± 0.05	**<0.001**	0.248	0.139	0.049	0.107	0.058	0.26	**0.001**	0.314	0.973
*Collinsella aerofaciens*	0.02 ± 0.00	0.02 ± 0.00	0.04 ± 0.0.01	0.02 ± 0.00	0.01 ± 0.01	0.301	0.024	0.200	0.037	0.118	0.055	0.047	0.185	0.112	0.462

Values are means ± SEM. Cecal microbiota data are shown as means ± SEM expressed as the relative abundance (%) of bacterial taxa per total bacteria (16S rRNA gene copies of microbial group/total bacteria 16S rRNA gene copies). Chow: chow control diet; HFS: high fat/high sucrose diet; F: prebiotic fibre; E: aerobic exercise. HDL: high density lipoprotein; LDL: low density lipoprotein; Chol: cholesterol. Trig: triglycerides.

### The combination of prebiotic fibre and aerobic exercise prevents increases in percent body fat in rats exposed to a HFS diet

Absolute percent body fat in rats in the HFS group was 11% higher than rats rats fed a standard chow control diet (p = 0.002, Fig. 2), and 8% higher than HFS fed rats that received prebiotic fibre supplementation and undertook aerobic exercise at the same time (HFS + F + E; p = 0.013). There were no statistically significant differences for percent body fat between the chow, HFS combined with prebiotic fibre supplementation (HFS + F), HFS combined with aerobic exercise (HFS + E), and HFS + F + E group animals (Fig. 2, Table 1). Additionally, percent body fat had a positive association with knee joint damage (Table 2).

**Table 2:** Relationship between knee joint damage (Modified Mankin Score), rat metabolic profile and cecal microbiota.

	Knee Joint Damage			Knee Joint Damage	
	r	p-value		r	p-value
Percent body fat	0.327	**0.014**	*Bacteroides/Prevotella*	−0.328	**0.013**
SF: leptin	0.451	**0.001**	*Bifidobacterium*	−0.320	**0.016**
Serum: leptin	0.389	**0.003**	*Enterobacteriaceae*	0.210	0.121
Endotoxin	0.455	**<0.001**	*Lactobacillus*	−0.125	0.359
CISI	−0.327	**0.010**	*C*. *coccoides* (cluster XIV)	−0.020	0.885
Total cholesterol	0.343	**0.012**	*C*. *leptum* (cluster IV)	0.436	**0.001**
HDL-cholesterol	0.230	0.087	C. cluster I	0.226	0.094
LDL-cholesterol	0.264	0.052	C. cluster XI	0.206	0.127
Non-HDL cholesterol	0.399	**0.015**	*Roseburia*	−0.294	**0.028**
Triglycerides	0.546	**<0.001**	*Methanobrevibacter*	0.237	0.079
Ratio: Total Chol/HDL	0.220	0.116	*Akkemansia muciniphila*	0.307	**0.021**
Ratio: Trig/HDL	0.330	**0.017**	*Faecalibacterium prausnitzii*	0.457	**<0.001**
			*Collinsella aerofaciens*	0.008	0.953

r: Pearson’s correlation coefficient; SF: synovial fluid; CISI: composite insulin sensitivity index; HDL: high density lipoprotein; LDL: low density lipoprotein; Chol: cholesterol. Trig: triglycerides.

On average, body mass in the beginning of the experimental protocol was 469 g per rat, and it increased for all groups of rats during the experimental period. HFS fed rats had an increase in body mass of 56% from the baseline measurements, while chow, HFS + F, HFS + E, and HFS + F + E rats increased 36%, 41%, 34%, and 32%, respectively (F_(4,51)_ = 13.830, p < 0.001).

### Prebiotic fibre and aerobic exercise prevent increases in local and systemic leptin concentration levels in rats exposed to a HFS diet

Rats in the HFS group had higher leptin levels in knee synovial fluid than did rats in the chow, HFS + F, HFS + E, and HFS + F + E groups (p = 0.001, p = 0.034, p = 0.040, p = 0.014, respectively), indicating that the interventions prevented the increases in synovial fluid leptin levels detected in the untreated obese animals (Table 1). Serum leptin levels exhibited the same pattern as the synovial fluid leptin. The HFS group animals had higher serum leptin levels than did the chow group rats (p = 0.006). Prebiotic fibre, aerobic exercise, and the combination of both prevented increases in serum leptin in rats on the HFS diet (p = 0.010, p = 0.038, and p = 0.019, respectively, Supplementary Material Table 2). Additionally, synovial fluid and serum leptin exhibited a positive association with knee joint damage (Table 2).

Overall, the other 26 biomarkers assessed in the synovial fluid and serum did not differ between groups (Supplementary Material Table 2).

### Prebiotic fibre and aerobic exercise prevent decreases in insulin sensitivity of rats exposed to a HFS diet

HFS fed rats had decreased whole body insulin sensitivity compared to rats fed a chow control diet (p = 0.001). Prebiotic fibre, aerobic exercise, and the combination of the two normalized insulin sensitivity to levels found in the chow group rats (p = 0.004, p = 0.011, p = 0.003, compared to HFS group, respectively; Fig. 2, Table 1). Insulin sensitivity exhibited a negative association with knee joint damage (Table 2).

### Prebiotic fibre and aerobic exercise improve serum lipid profile of rats fed a HFS diet

The HFS diet led to increased serum total cholesterol, HDL-cholesterol, triglycerides, and the triglycerides to HDL-cholesterol ratio compared to rats fed a chow diet (Fig. 2). The combined intervention of prebiotic fibre and exercise prevented these increases in serum total cholesterol and HDL-cholesterol levels (Table 1, Fig. 2). Prebiotic fibre supplementation, exercise, and the combination of the two prevented increases in serum triglyceride levels and increases in the serum triglycerides to HDL-cholesterol ratio (Fig. 2, Table 1). Triglycerides levels exhibited a strong positive association with knee joint damage, while total cholesterol and non-HDL-cholesterol exhibited a moderate positive association with knee joint damage, and HDL- and LDL-cholesterol exhibited weak positive associations with knee joint damage (Table 2).

### Prebiotic fibre and aerobic exercise prevent increases in serum endotoxin in rats exposed to a HFS diet

The HFS diet led to increased serum endotoxin levels (p = 0.002) compared to chow fed rats. Prebiotic fibre, aerobic exercise, and the combination of exercise and prebiotic fibre prevented these increases (p < 0.001, p < 0.001, and p < 0.001, respectively), maintaining values similar to those in chow fed control rats (Fig. 2b, Table 1). Endotoxin levels exhibited a positive association with knee joint damage (Table 2).

### Prebiotic fibre, but not exercise, prevents microbial dysbiosis in rats fed a HFS diet

**Figure 3:**
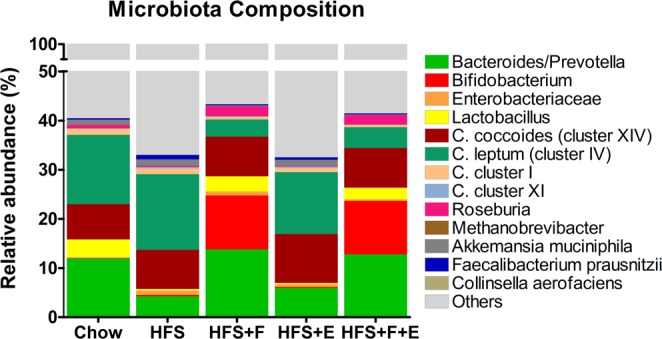
Microbiota composition. Cecal matter microbiota composition at the end of the experimental protocol. Values are mean relative abundance of cecal microbiota. Microbial abundance was measured as 16S rRNA gene copies per 20 ng DNA and reported here as relative abundance (%) of bacterial taxa per total bacteria. Chow: chow control diet; HFS: high fat/high sucrose diet; F: prebiotic fibre; E: aerobic exercise.

The cecal microbiota composition of the HFS rats showed a decrease in *Bacteroides/Prevotella* (p = 0.048), *Lactobacillus* (p < 0.001), and *Faecalibacterium prausnitzii* (p = 0.024) compared to chow fed rats that was prevented by prebiotic fibre supplementation, but not by exercise; and an increase in *Enterobacteriaceae* (p = 0.010), that was prevented by the combination of prebiotic fibre and exercise (Fig. 3, Table 1). Prebiotic fibre supplementation, and the combination of prebiotic fibre and aerobic exercise, shifts the microbiota towards a decrease in *Clostridium leptum (cluster IV)*, *Clostridium cluster I*, *Methanobrevibacter*, and *Akkemansia muciniphila*; and an increase in *Bifidobacterium* and *Roseburia* compared to the cecal microbiota of HFS rats (p < 0.05, Fig. 3, Table 1). *Bacteroides/Prevotella*, *Bifidobacterium and Roseburia* exhibited a negative association with knee joint damage, while *Clostridium leptum (cluster IV)*, *Akkemansia muciniphila* and *Faecalibacterium prausnitzii* exhibited a positive association with knee joint damage (Table 2).

## Discussion

**Figure 4:**
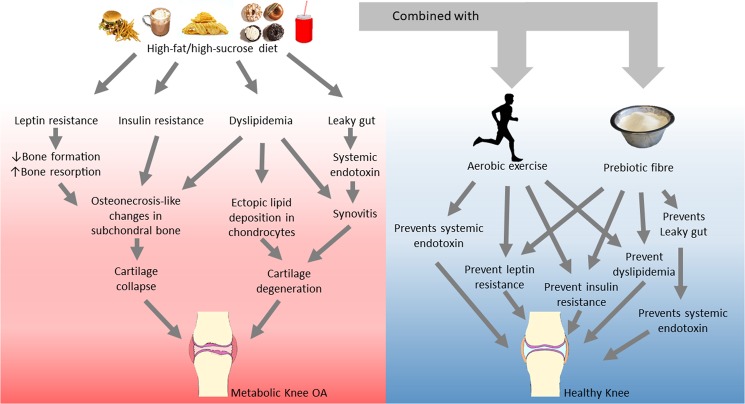
Diagram summarizing the findings of the present study. Model for the link between HFS diet and metabolic knee OA, and the pathways which prebiotic fibre and aerobic exercise might have activated to prevent damage of the knee joint in Sprague Dawley rats fed a HFS diet. Photographs are under Public Domain or CC0 Creative Commons License.

We showed that prebiotic fibre supplementation and aerobic exercise protect against the development of knee joint damage in rats fed a HFS diet. The maintenance of knee joint integrity achieved with prebiotic fibre and aerobic exercise was accompanied by a maintenance of metabolic and gut microbiota homeostasis. It appears that the obesity-related metabolic dysregulation and gut microbiota dysbiosis might be strongly linked to the metabolic OA phenotype in the Sprague Dawley rat model (see schematic in Fig. 4).

Majority of the damage detected in the knee joint of HFS fed rats occurred in the subchondral bone, and the structural changes observed in this study are similar to changes reported in an emu femoral head model of osteonecrosis^23^, suggesting a potential initiating role for subchondral bone changes in OA pathogenesis associated with the HFS diet. Such a role has been advanced previously in the literature as a result of reduced blood supply and compromised nutrient exchange to the bone^2,3^.

The metabolic OA phenotype is associated with hyperglycaemia, insulin resistance, and leptin resistance, which in turn are linked to bone formation/structure^2,3,24–27^. Hyperglycaemia and insulin resistance combined with increased leptin levels may have resulted in some of the changes observed in the subchondral bone of the HFS animals. Additionally, altered lipid metabolism in patients has also been associated with OA development in metabolic disease^3,28^. Rats fed the HFS diet had an altered lipid metabolism, as indicated by the increase in serum triglyceride levels and serum triglycerides to HDL-cholesterol ratio, and we detected a strong association between triglycerides levels and knee joint damage (Table 2).

The prebiotic oligofructose fibre supplementation used in our study prevented dyslipidemia, thereby potentially contributing to the maintenance of knee joint homeostasis. It has been shown that drugs that lower lipid levels inhibit OA development in a mouse model of atherosclerosis^29^. Elevated plasma cholesterol levels trigger oxidative stress in mitochondria and degradative changes in chondrocytes that, in combination, are thought to lead to OA development and progression^30^. Moreover, mouse models of hypercholesterolemia-induced OA are characterized by changes in subchondral bone architecture that are rescued when lipid-lowering drugs are administrated^30^. It has been suggested that hypercholesterolemia induces oxidation and deposition of lipids in tissues, resulting in damage to cartilage similar to that seen in atherosclerosis^31^, while osteonecrosis might occur because of a narrowing of the vasculature by lipids.

Obesity has been associated with increased gut permeability, allowing bacterial lipopolysaccharides from Gram-negative bacteria to translocate into the blood (endotoxemia), triggering systemic inflammation and insulin resistance^21,32^. It has been shown that improvements in glucose tolerance in rodents after prebiotic fibre supplementation are related to changes in the microbiota^33,34^. We made similar observations regarding changes in microbiota. Increases in the inflammation-associated *Enterobacteriaceae*^35^ were prevented by the combination of prebiotic fibre and exercise only. Prebiotic fibre supplementation also increased *Bifidobacterium* compared to HFS fed rats. *Bifidobacterium* has been shown to decrease intestinal permeability, upregulate expression of tight junction proteins and reduce IL-6 and TNF-α in cell lines and in rodents^36^. Given that oligofructose is known to increase the abundance of *Bifidobacterium*, it is possible that the prebiotic fibre reduced serum endotoxin levels via this mechanism. Furthermore, certain lactobacilli strains have been associated with health-promoting properties, including attenuated weight gain and improved gut barrier function^37^, which suggest that the HFS fed animals may have increased gut permeability due in part to the decrease in lactobacilli abundance.

Moreover, in agreement with our finding, it has been shown in a mouse model of high-fat diet–induced obesity that prebiotic fibre supplementation has a protective effect against trauma- induced OA. Manipulation of the gut microbiota is thought to represent a viable disease-modifying therapeutic strategy for OA associated with obesity^38^.

The aerobic exercise used in this study consisted of a progressive treadmill training of up to 150 minutes week. This amount of exercise corresponds to the recommended minimal physical activity guidelines for humans^39^, and has been shown to be safe for Sprague Dawley knees^40^. Aerobic exercise in our rats was found to modulate insulin and leptin levels, similar to what has been observed for the prebiotic fibre supplementation. Aerobic exercise in humans is also associated with decreased plasma leptin and insulin levels^41^. Thus, maintenance of insulin homeostasis in the exercising rats may have prevented increases in leptin and leptin resistance in rats fed a HFS diet, despite similar body fat percentages in HFS + E and HFS rats. Therefore, aerobic exercise may have helped maintain bone turnover homeostasis, thereby preventing the development of OA-like damage in the knees of rats fed the HFS diet.

Aerobic exercise also prevented increases in serum triglyceride levels and serum triglycerides to HDL-cholesterol ratio observed in rats exposed to a HFS diet. These results agree with findings from human studies. For example, it has been shown that there is a dose-response relationship between physical activity and improvements in triglycerides and HDL-cholesterol levels in previously sedentary people^42^. However, the mechanisms underlying these improvements are unclear. By preventing dyslipidemia, aerobic exercise may have contributed to the maintenance of knee joint integrity in the HFS rats.

Aerobic exercise also prevented endotoxemia in rats fed a HFS diet. However, the pathway might not be the same as that suggested for the prebiotic fibre intervention. Endotoxin, an enteric bacteria-derived molecule, is recognized by Toll-like receptors (TLRs) on immune cells, adipocytes, and cells in several organs, such as Kupffer cells in the liver, and when in excess, triggers an inflammatory response^43^. The capacity of Kupffer cells to remove endotoxin might be important in the prevention of metabolic knee OA. Aerobic exercise has been shown to improve the removal of endotoxins by Kupffer cells^44^, thereby contributing to the reduction of endotoxemia in rats fed a HFS diet, and possibly preventing the OA-like damage observed in the rat knee.

While prebiotic fibre supplementation and aerobic exercise prevented the development of knee damage, and affected the metabolic outcome variables to a similar extent, exercise did not affect the gut microbiota, while the prebiotic fibre intervention did. Therefore, it appears that the prebiotic intervention affected the microbiota, and as a consequence, the host secondarily via the fermentation of the prebiotic fiber, short chain fatty acids^21^ (not assessed in our study). In contrast, the exercise protocol primarily affected host parameters to diminish the impact of the HFS diet and the altered microbiota. Thus, the exercise protocol appeared to impact the metabolic profile and the gut leaky syndrome by increasing the uptake of endotoxins by Kupffer cells^44^. Aerobic exercise combined with prebiotic fibre supplementation was not more effective as each intervention alone (Fig. 4). Therefore, it appears that the mechanisms involved for each intervention complement each other but are not additive. Therefore, the impact of the HFS diet on a sedentary host is different than its impact on a host that is exercising and enhancing the resistance of host systems. Exercise is expect to contribute to tissue repair, enhance the metabolism of glucose and fat, and enhance the release of exercise-associated regulatory molecules from the brain to counteract diet-induced factors^45^. It is known that mechanical loading of tissues, such as cartilage, ligaments, tendons, and skin, can lead to resistance to catabolic mediators^46^ or their expression^47^. Therefore, exercise-induced tissue loading may result in the maintenance of tissue integrity. The non-exercised rats in this study had reduced tissue loads compared to exercised rats, and their systems might have been at an increased risk for alteration caused by the HFS diet. It appears that exercise and prebiotic fibre supplementation impact HFS fed differently. Providing both interventions simultaneously may lead to a reinforcement of resistance to the adverse effects of the HFS diet over 12 weeks.

In summary, a HFS diet led to development of knee joint damage in rats that was associated with changes in the metabolic profile in these animals. We provide novel information on the prevention of metabolic OA using a prebiotic fibre and/or an aerobic exercise intervention (Fig. 4). We suggest that the preventive effects of the prebiotic fibre and aerobic exercise interventions are associated with the leptin and insulin pathways, as well as with lipid metabolism and the leaky gut syndrome. Effectively addressing the prevention of metabolic osteoarthritis will require the engagement of public health programmes. Low cost, non-invasive treatments strategies, such as prebiotic fibre supplementation and aerobic exercise should be a priority.

## Methods

### Animals and intervention protocol

Forty-eight male, 12-week old, Sprague Dawley rats, fed a high-fat/high-sucrose diet (HFS, 20% of total weight as fat, 50% sucrose, 20% protein, and 10% from fibre and micronutrients; custom Diet #102412, Dyets, United States) were randomized into a sedentary (HFS, n = 12), aerobic exercise (HFS + E, n = 12), prebiotic fibre supplementation (HFS + F, n = 12), or aerobic exercise combined with prebiotic fibre supplementation (HFS + F + E, n = 12) group for 12 weeks. Eight chow-fed (standard chow diet control group, 5% of total weight as fat, 47.5% carbohydrates (only 4% from sucrose), 25% protein, 12.5% from fibre and micronutrients, and 10% moisture; Lab Diet 5001, United States, n = 8), age- and sex-matched animals were included as controls. The aerobic exercise intervention consisted of a progressive moderate treadmill training program for 12 weeks, up to 30 minutes per day, 5 days a week as described previously (Supplementary Material Table 1)^40,48^. The prebiotic fibre was supplemented in the diet at a dose of 10% (wt/wt, Orafti P95, BENEO-Orafti, Germany)^18,49^. Calculation of sample size was performed using G*Power Software (version 3.0.10, Germany)^50^. Data for sample size calculations were based on a previous study with HFS and control rats^9^. All experiments were approved by the University of Calgary Life and Environmental Sciences Animal Care Committee, and all methods were conducted in accordance with the animal welfare regulations and guidelines at the University of Calgary.

### Body composition

Three days after completing the 12 week intervention protocol, rats were lightly anaesthetized with isoflurane and body composition was measured using Dual X-ray absorptiometry (DXA) with software for small animals (Hologic ODR 4500; Hologic, Bedford, MA, USA). The mean of three repeat scans for each animal were used for analysis.

Body mass was measured at the beginning of each week. Body mass for each animal was normalized to that of week 1 (familiarization week) and was expressed as the percent increase in body mass from that initial value.

### Blood glucose and insulin

One day after the end of the intervention period, following 16 h of food deprivation, rats were given an oral gavage of 2 g/kg glucose. Blood was collected at 0, 15, 30, 60, and 120 min post-gavage via tail nick in a chilled tube for insulin analysis. Blood glucose was measured immediately with a blood glucose meter (OneTouch Verio and Blood Glucose Monitoring System, Lifescan, Switzerland). Whole body insulin sensitivity was determined using proxy measures from the glucose tolerance tests (composite insulin sensitivity index – CISI)^51^.

### Blood lipid profile and endotoxin

One week prior to the start of the 12 week intervention protocol (acclimatization week), following 16 h of food deprivation, blood was collected via tail nick to evaluate the baseline values for cytokines/adipokines (details below). Four days after completing the 12 week intervention protocol, and following 16 h of food deprivation, rats were anaesthetized with isoflurane and cardiac blood samples were collected. Serum was analyzed for lipid profile (total cholesterol, LDL-cholesterol, HDL-cholesterol, and triglycerides) using colorimetric assays (Calgary Lab Service, Calgary, AB, Canada), cytokines/adipokines (details below), and for endotoxin. Concentration of blood endotoxin was measured using the Pierce LAL Chromogenic Endotoxin Quantification assay (Thermo Fisher Scientific, MA, USA) according to manufacturer directions.

### Blood and synovial fluid cytokines and adipokines

The right knee joints were opened to collect synovial fluid shortly after sacrifice using the Whatman chromatography paper method^52^. Samples were weighed, diluted and centrifuged^40^. Serum and synovial fluid cytokines and adipokines were quantified using a Rat 27 Multiplex Discovery Assay with Luminex®xMAP technology (Eotaxin, EGF, Fractalkine, IL-1α, IL-1β, IL-2, IL-4, IL-5, IL-6, IL-10, IL-12(p70), IL-13, IL-17A, IL-18, IP-10/CXCL10, GRO/KC, IFN-γ, TNF-α, G-CSF, GM-CSF, MCP-1, leptin, LIX, MIP-1α, MIP-2, RANTES, VEGF; Eve Technologies, AB, Canada).

### Cecal matter microbiota

Microbiota analysis was carried out using 16rRNA qPCR as per our previous work^53^. Cecal samples were collected at euthanasia for cecal microbiota analysis. DNA was extracted from the cecal samples using the MP Biomedicals Fast DNA Spin Kit for Feces (MP Biomedicals, Lachine, QC, Canada). Amplification and detection were conducted in 96-well plates with SYBR Green 2 × qPCR Master Mix (BioRad). Purified template DNA from reference strains was used to generate standard curves for each primer set using 10-fold serial dilutions of DNA. Standard curves were normalized to copy number of 16S rRNA genes using reference strain genome size and 16S rRNA gene copy number values. Total bacteria were also measured. Primer selection for qPCR was based on providing broad coverage of the total microbial signal in rats and those microbes of relevance to an obese rat model^53^.

### Knee joint histology

The left knee was harvested by cutting the femur and tibia/fibula 2 cm above and below the joint line, respectively. Muscles were removed and joints were fixed in a 10% neutral buffered formalin solution for 14 days at room temperature. Knees were then decalcified, dehydrated in a graded series of alcohols, embedded in paraffin wax, and stored at room temperature until sectioning^40^. Serial sagittal plane sections of 10 µm thickness were obtained, and mounted. Subsequently, every second section was stained sequentially with haematoxylin, fast green and safranin-O^40^. Two independent, blinded graders scored all histological sections using a Modified Mankin Histology Scoring System^54^, and the Osteoarthritis Research Society International’s (OARSI) histologic^55^ sub-scores for bone changes, synovial thickening and menisci for each joint. Only the worst defect quantified per joint was used for the statistical analyses. The total Modified Mankin Score for each animal was defined as the sum of all individual OARSI sub-scores and the Modified Mankin Score.

### Statistical analysis

All data are presented as means ± 1 SEM. Levene’s test for equality of variance was conducted on all outcomes. A one way ANOVA with the five experimental groups as the main factor was performed using Bonferroni post hoc testing to determine differences between groups. A 2-way ANOVA was used to determine the main effects of prebiotic fibre supplementation, aerobic exercise, and their interaction, on the HFS diet. Changes in serum cytokines and adipokines from pre- to post-intervention were determined using a repeated measures ANOVA with time as the within-subject factor and experimental grouping as the between-subject factor. To further explore the changes in serum cytokines and adipokines pre- and post-intervention, data were normalized relative to the pre-intervention values, and a one way ANOVA with experimental grouping as the main factor was performed using Bonferroni post hoc testing. To measure the association between knee joint damage, the metabolic variables, and cecal microbiota data, the Pearson Correlation Coefficient was calculated. Analyses were done using SPSS V22.0 software (SPSS, Chicago, IL, USA). Data were considered statistically significant at p < 0.05, two-tailed.

## Supplementary information

Supplementary Material
