# Supplementary material for: Protective effect of prebiotic and exercise intervention on knee health in a rat model of diet-induced obesity
Source: Sci Rep. 2019 Mar 7;9:3893. doi: 10.1038/s41598-019-40601-x (PMC6405910; doi:10.1038/s41598-019-40601-x)
Supplement: Supplementary file 1 — Supplementary Material [file 41598_2019_40601_MOESM1_ESM.pdf]

SUPPLEMENTARY INFORMATION

Protective effect of prebiotic and exercise intervention on knee health in a rat model of diet-induced obesity

Jaqueline Lourdes Rios MSc, PT [1, 2,*], **Marc R. Bomhof** PhD [1, 3], **Raylene A. Reimer** PhD [1,2,4], **David A. Hart** PhD [1,2], **Kelsey H. Collins** PhD [1, 2], **Walter Herzog** PhD [1, 2]

[1]Human Performance Laboratory, Faculty of Kinesiology, University of Calgary, Calgary, AB, Canada;

[2]McCaig Institute for Bone and Joint Health, University of Calgary, Calgary, AB, Canada;

[3]Department of Kinesiology & Physical Education, University of Lethbridge, Lethbridge, AB, Canada;

[4]Department of Biochemistry and Molecular Biology, University of Calgary, Calgary, AB, Canada

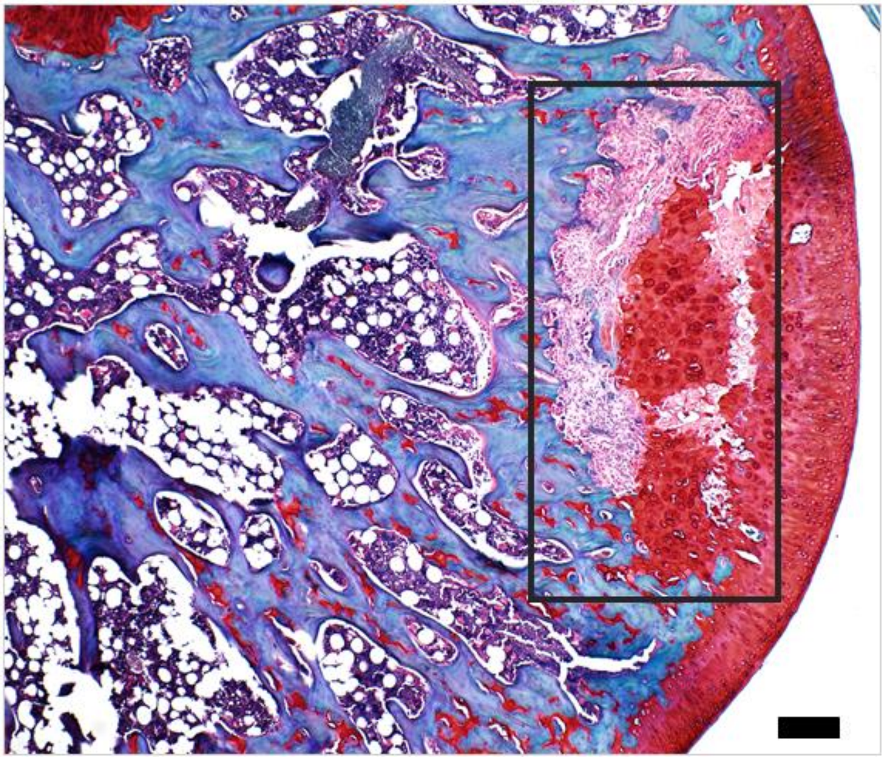

Supplementary Figure 1: Knee joint: medial/posterior femur. This image represents the knee joint of the 3 rats fed a HFS diet that did not have the collapse of the cartilage. Inside the rectangle you can notice changes in the subchondral bone that might predispose the further cartilage collapse (black line, bar = 200 µm).

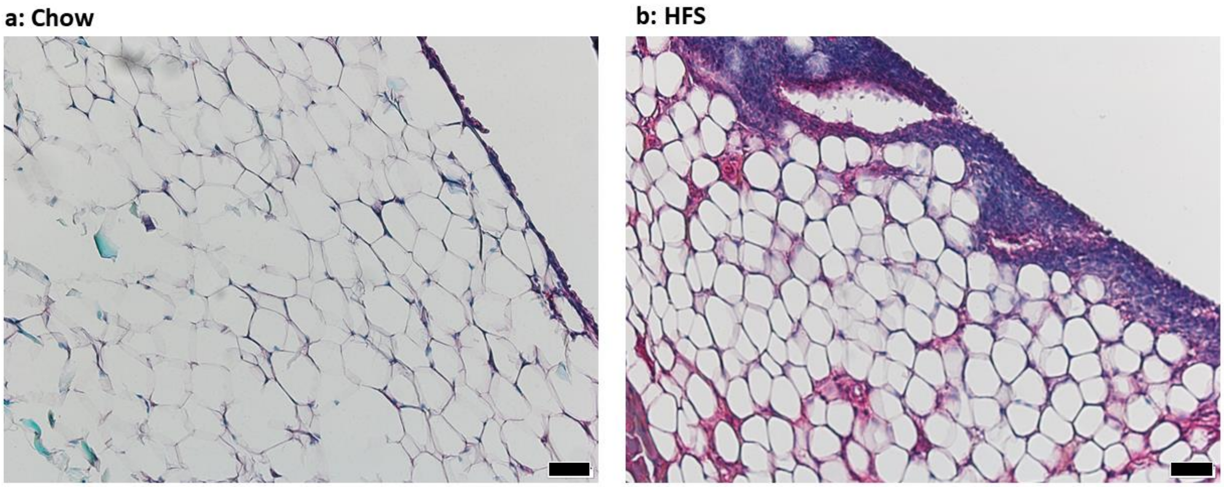

Supplementary Figure 2: Thickening of synovium was presented in HFS sedentary rats but not in Chow rats (black line, bar = 50 μm).

Supplementary Table 1. Twelve Week Progressive Treadmill Training Program.

Week	Speed m/min	Moderate Duration		
		Sessions/week	Sessions/day	Training Time (min)
**1**	0	5	1	10
**2**	15	5	1	20
**3**	20	5	1	30
**4**	22.5	5	1	30
**5-12**	25	5	1	30
Total travelled distance			37.9 km<br>23.6 miles	

Week 1 was a familiarization week. Rats were placed on the treadmill for 10 minutes, with the grid shock turned on, but with the treadmill belt not running. HFS diet and fiber supplementation started on week 1, after the body mass measurement.

Supplementary Table 2. Serum 27 cytokine/adipokine profile for rats in the Chow, HFS, HFS+F, HFS+E, HFS+F+E groups before and at the end of the experimental period.

	Chow		HFS		HFS+F		HFS+E		HFS+F+E		Repeated measures ANOVA					
											Time		Group		Time *Group	
(unit)	Pre	Post	Pre	Post	Pre	Post	Pre	Post	Pre	Post	p-value	partial η^2	p-value	partial η^2	p-value	partial η^2
G-CSF	44.8 ± 4.2	34 ± 8.4	40.5 ± 3.1	40.6 ± 4.3	41.6 ± 3.3	33.2 ± 6.0	41.8 ± 3.9	36.7 ± 5.5	40.2 ± 4.3	33.7 ± 4.2	0.051	0.084	0.855	0.029	0.82	0.034
Eotaxin	5.6 ± 0.5	5.4 ± 0.9	5.4 ± 0.5	6.0 ± 0.8	3.9 ± 0.3	4.4 ± 1.0	4.5 ± 0.6	4.4 ± 0.8	5.6 ± 0.5	4.7 ± 0.4	0.905	<0.001	0.221	0.133	0.873	0.030
IL-1a	50.8 ± 10.7	36.6 ± 15.8	70.1 ± 12.2	31.6 ± 9.0	129.1 ± 66.5	31.3 ± 7.5	57.9 ± 11.2	28.4 ± 9.5	57.5 ± 16.6	16.0 ± 3.7	**0.008**	0.289	0.832	0.065	0.953	0.031
Leptin	5288 ± 787	8692 ± 1308	6358 ± 810	33005 ± 4849	9202 ± 1042	26926 ± 5103	11827 ± 1586	21002 ± 3212	8617 ± 2232	15278 ± 2182	**<0.001**	0.524	**0.004**	0.258	**<0.001**	0.328
MIP-1a	11.3 ± 1.7	7.5 ± 0.7	12.9 ± 1.4	9.3 ± 0.7	14.7 ± 1.8	10.2 ± 1.1	13.1 ± 1.3	7.6 ± 1.1	11.4 ± 1.2	7.4 ± 0.7	**<0.001**	0.380	0.101	0.141	0.933	0.016
IL-4	3.3 ± 0.6	4.1 ± 1.5	4.4 ± 0.7	10.4 ± 4.1	2.7 ± 0.5	7.4 ± 3.3	4.6 ± 2.0	4.2 ± 1.0	2.9 ± 0.6	2.3 ± 0.6	0.107	0.061	0.238	0.121	0.235	0.121
IL-1B	69.5 ± 37.8	37.2 ± 8.0	94.2 ± 24.3	153.0 ± 42.2	86.7 ± 29.1	67.3 ± 25.6	93.1 ± 36.8	122.3 ± 44.9	55.8 ± 14.1	42.4 ± 13.7	0.721	0.003	0.240	0.100	0.144	0.123
IL-2	46.0 ± 5.1	50.9 ± 6.9	49.9 ± 3.7	66.0 ± 6.2	47.7 ± 7.0	55.8 ± 9.9	49.6 ± 5.7	46.1 ± 7.8	54.2 ± 4.4	45.8 ± 4.2	0.256	0.026	0.628	0.050	0.124	0.132
IL-13	5.6 ± 1.6	8.4 ± 2.9	6.4 ± 1.4	6.5 ± 1.6	3.0 ± 1.0	5.1 ± 1.9	4.4 ± 1.2	5.9 ± 2.1	5.6 ± 1.1	2.7 ± 0.7	0.888	0.001	0.340	0.149	0.555	0.102
IL-10	55.0 ± 29.7	35.7 ± 10.2	75.0 ± 14.3	118.8 ± 27.7	68.8 ± 18.2	61.2 ± 20.5	77.8 ± 23.5	90.2 ± 30.8	49.0 ± 10.4	38.2 ± 12.0	0.667	0.004	0.210	0.107	0.157	0.120
IL-12	151.2 ± 15.2	176.8 ± 20.8	175.3 ± 8.6	200.0 ± 10.9	141.6 ± 13.6	162.6 ± 14.0	152.1 ± 19.7	141.9 ± 20.4	161.3 ± 8.7	153.7 ± 14.6	0.231	0.028	0.097	0.140	0.502	0.062
IL-5	36.5 ± 3.6	38.1 ± 2.8	43.1 ± 1.8	44.9 ± 2.6	39.4 ± 2.6	35.7 ± 3.7	41.7 ± 3.4	34.1 ± 2.8	42.6 ± 2.7	36.5 ± 2.2	0.138	0.043	0.114	0.133	0.365	0.08
IL-17A	10.1 ± 2.0	11.9 ± 2.0	11.1 ± 1.4	18.4 ± 2.8	9.7 ± 1.3	10.6 ± 2.0	11.1 ± 1.4	9.2 ± 1.5	12.3 ± 1.4	10.4 ± 1.0	0.207	0.031	0.095	0.141	**0.021**	0.199
IL-18	274.7 ± 32.8	133.4 ± 14.3	236.7 ± 17.9	168.4 ± 19.8	301.9 ± 45.5	215.3 ± 57.2	237.7 ± 25.2	124.1 ± 26.8	225.1 ± 22.2	109.3 ± 15.9	**<0.001**	0.408	0.095	0.141	0.752	0.036
MCP-1	508.6 ± 79.1	459.0 ± 50.3	602.4 ± 56.8	603.4 ± 53.9	586.5 ± 52.3	468.0 ± 41.6	558.6 ± 48.1	351.8 ± 51.2	586.1 ± 41.1	485.1 ± 45.0	**0.001**	0.197	0.158	0.124	0.14	0.129
IP-10	188.5 ± 17.0	150.5 ± 13.6	226.9 ± 12.7	206.0 ± 15.6	248.5 ± 19.3	194.0 ± 18.0	248.2 ± 21.2	196.6 ± 26.7	210.0 ± 16.2	162.7 ± 22.0	**<0.001**	0.28	0.119	0.132	0.778	0.033
VEGF	18.3 ± 5.2	14.5 ± 3.4	21.4 ± 2.7	24.8 ± 2.8	28.8 ± 4.3	25.7 ± 3.8	26.0 ± 4.0	19.3 ± 3.2	21.3 ± 2.7	14.4 ± 2.7	**0.048**	0.086	0.076	0.171	0.296	0.104
Fractalkine	32.9 ± 4.3	30.1 ± 1.5	39.1 ± 2.5	36.6 ± 3.0	48.0 ± 3.8	41.4 ± 3.1	43.3 ± 5.1	30.4 ± 4.4	37.6 ± 2.8	30.9 ± 2.8	**0.001**	0.204	**0.044**	0.172	0.332	0.085
LIX	1879 ± 258	1298 ± 182	2108 ± 158	1701 ± 204	2122 ± 262	1291 ± 165	2334 ± 306	1219 ± 218	1976 ± 171	1266 ± 178	**<0.001**	0.370	0.654	0.046	0.496	0.063
TNFa	9.9 ± 2.0	11.8 ± 2.2	10.8 ± 1.1	14.3 ± 1.5	8.0 ± 1.1	10.2 ± 1.7	9.3 ± 1.3	6.8 ± 1.1	11.3 ± 1.1	9.5 ± 1.2	0.669	0.004	**0.048**	0.178	0.102	0.146
RANTES	647.0 ± 117.1	396.9 ± 69.6	1282 ± 298	1145 ± 318	1376 ± 381	550 ± 91	1604 ± 468	810 ± 284	889 ± 197	487 ± 82	**0.002**	0.179	0.192	0.111	0.422	0.072

Values are means ± SEM. Chow: chow control diet; HFS: high fat/high sucrose diet; F: prebiotic fibre; E: aerobic exercise.

GM-CSF, IL-6, IFNy, GRO/KC, MIP-2, and EGF were excluded from analysis due to extrapolated values and/or values out of validated range.
